# Mechanisms of aging in senescence-accelerated mice

**DOI:** 10.1186/gb-2005-6-6-r48

**Published:** 2005-06-01

**Authors:** Todd A Carter, Jennifer A Greenhall, Shigeo Yoshida, Sebastian Fuchs, Robert Helton, Anand Swaroop, David J Lockhart, Carrolee Barlow

**Affiliations:** 1 The Salk Institute for Biological Studies, La Jolla, CA 92037, USA; 2 Department of Ophthalmology and Visual Sciences, University of Michigan, Ann Arbor, MI 48105, USA; 3 Department of Human Genetics, University of Michigan, Ann Arbor, MI 48105, USA; 4 Ambit Biosciences, San Diego CA 92121, USA; 5 Current address: BrainCells Inc., 10835 Road to the Cure, San Diego, CA 92121, USA

## Abstract

**Background:**

Progressive neurological dysfunction is a key aspect of human aging. Because of underlying differences in the aging of mice and humans, useful mouse models have been difficult to obtain and study. We have used gene-expression analysis and polymorphism screening to study molecular senescence of the retina and hippocampus in two rare inbred mouse models of accelerated neurological senescence (SAMP8 and SAMP10) that closely mimic human neurological aging, and in a related normal strain (SAMR1) and an unrelated normal strain (C57BL/6J).

**Results:**

The majority of age-related gene expression changes were strain-specific, with only a few common pathways found for normal and accelerated neurological aging. Polymorphism screening led to the identification of mutations that could have a direct impact on important disease processes, including a mutation in a fibroblast growth factor gene, *Fgf1*, and a mutation in and ectopic expression of the gene for the chemokine CCL19, which is involved in the inflammatory response.

**Conclusion:**

We show that combining the study of inbred mouse strains with interesting traits and gene-expression profiling can lead to the discovery of genes important for complex phenotypes. Furthermore, full-genome polymorphism detection, sequencing and gene-expression profiling of inbred mouse strains with interesting phenotypic differences may provide unique insights into the molecular genetics of late-manifesting complex diseases.

## Abstract 2

Gene-expression analysis and polymorphism screening to study molecular senescence of the retina and hippocampus in two rare inbred mouse models of accelerated neurological senescence as well as a related and an unrelated normal strain showed that most age-related gene expression changes were strain-specific.

## Background

Aging is defined by an increase in the probability of death over time associated with characteristic changes in phenotype [1]. Changes in the global control of transcription have been directly implicated in the aging process in yeast, and increased histone deacetylation activity (a process involved in chromatin silencing) results in extended life span in *Caenorhabditis elegans *[2-4]. Genomic instability has also been implicated as a causative agent in cellular senescence in mammals. This relationship between genomic instability and aging in mammals is supported by work demonstrating a correlation between senescence and the loss of ribosomal DNA, increases in chromosomal abnormalities and telomere shortening [1,5-7]. In addition, certain mutations in humans can accelerate aging-specific events, resulting in progeric diseases that include Hutchinson-Gilford syndrome, Werner syndrome, Cockayne syndrome and xeroderma pigmentosum [8-10]. Except for Hutchinson-Gilford syndrome, each of these disorders results from mutations in DNA repair proteins, suggesting that a stochastic build-up of errors in DNA could form the basis for some common traits of aging. Recent studies have indicated that Hutchinson-Gilford syndrome is caused by specific mutations in lamin A, a gene involved in structural integrity of the nuclear membrane [11,12]. Interestingly, some genetic disorders that exhibit aspects of accelerated senescence also demonstrate genomic instability, including several mentioned above as well as ataxia-telangiectasia and Bloom's syndrome [13-17].

While single-gene progerias can provide insight into age-related processes, most patients exhibit only a subset of the phenotypes associated with aging. Thus, the process may be fundamentally different from normal aging, which involves multiple events and tissues. To complement studies of single-gene progerias and other models of mammalian senescence, we have chosen to study a more complex model of aging: the senescence-accelerated mouse (SAM) strains. The senescence-accelerated mice are a collection of inbred mouse strains developed as models of accelerated aging, and include nine short-lived, senescence-accelerated mouse prone strains (SAMP) and three longer lived control strains designated senescence-accelerated mouse resistant (SAMR) [18]. The SAMP strains exhibit several features that make them interesting models of human aging, including age-associated early onset of senile amyloidosis, degenerative arthropathy, cataracts, osteoporosis and osteoarthritis, reduced fecundity and early loss of fertility [18-20]. Mapping studies have been limited to microsatellite haplotype analyses characterizing the genetic relationships between the SAM strains [21]. In addition, there is currently no genome sequence available for these strains, making it difficult to use comparative genomics to identify genetic differences responsible for their phenotypic differences. Furthermore, the strains involved in these studies require continual trait-based selection to maintain the phenotype. As standard quantitative trait locus mapping approaches would be extremely difficult with such strains, we sought to test the hypothesis that gene-expression profiling combined with candidate gene sequencing would lead to the identification of mutations and/or expression changes that track with the strain-specific phenotypes, thereby allowing us to identify relevant pathways and generate candidate genes for future experiments.

Our study focused on the identification of genes involved in neurological aspects of aging, using two SAMP strains: SAMP8/Ta (S8) and SAMP10//Ta (S10), and two control strains: the related SAMR strain SAMR1TA (SR1) and a commonly used inbred mouse strain C57BL/6J (B6J). The S8 and S10 strains exhibit age-related behavioral and neuropathological phenotypes, in addition to osteoporosis and premature loss of fertility, that make them particularly useful models of human neurological aging [22-25]. These phenotypes include deficits in learning and memory, emotional disorders and abnormal circadian rhythms [18,26]. S8 mice also develop a severe age-related impairment in acquisition and retention of the passive avoidance response, as well as a reduced-anxiety behavior [23,27]. Old S10 mice exhibit behavioral depression on tail suspension and forced swimming tests [23]. A unique pathological feature of senescence in S10 mice is an age-related atrophy of the brain [28]. Neuron shrinkage and degeneration in S10 mice result in progressive decrease of mean brain weight beginning at 4 months of age [28]. In addition to the neurobehavioral and physiological phenotypes, S8 mice demonstrate an age-related degeneration of the retinal pigment epithelium-Bruch's membrane-choriocapillaris complex, and a degeneration of receptor cells and ganglion neurons in the retina suggestive of age-related macular degeneration in humans [29].

The S8 and S10 strains are also interesting in that although inbred, trait-based selection is necessary to maintain the phenotype of the age-associated disorders over generations [30]. Thus, while the phenotypes are heritable, they are clearly part of a complex trait that probably involves the interaction of multiple genes and/or alleles, suggesting that these strains may better model the processes associated with mammalian aging than single-gene progeria models.

To explore the events involved in the molecular senescence of the mammalian brain, we established and aged the colonies, verified the phenotypes, and performed gene-expression analysis of the retina and hippocampus in S8 and S10 mice using oligonucleotide microarrays [31]. As a control, we studied the SAMR strain SR1. All SAM strains were originally derived from the AKR/J inbred mouse strain, but SR1 demonstrates a longer life span and lacks the accelerated senescence that is a hallmark of the SAMP strains. Furthermore, we also analyzed the gene-expression data in the context of an additional, unrelated inbred mouse strain, B6J, to distinguish strain-background specific (AKR-specific) from more general changes of the aging transcriptome. Finally, we took advantage of a focused polymorphism screen to identify two genes harboring mutations in the SAMP strains that may play important roles in their accelerated-aging phenotypes.

## Results

### Verification of phenotype in SAM Strains

**Figure 1:**
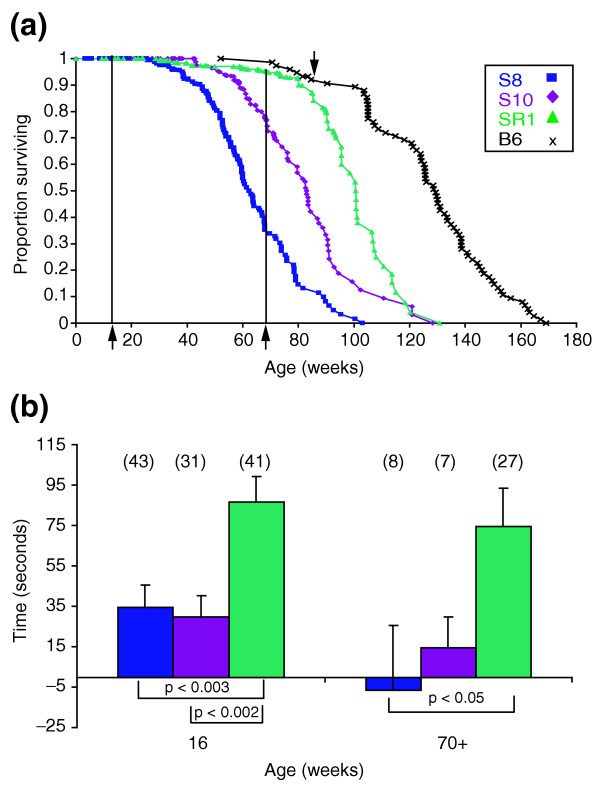
S8 and S10 mice exhibit accelerated-senescence phenotypes. **(a) **Proportion of surviving S8 mice (blue squares, *n *= 237), S10 mice (purple diamonds, *n *= 169), SR1 mice (green triangles, *n *= 189) and B6 mice (black crosses, *n *= 75, data from Pugh *et al*. [32]). Using a Kaplan-Meier survival analysis, the S8 and S10 survival profiles are significantly different from SR1 (*p *< 0.0001 and *p *< 0.0015, respectively) by the Mantel-Cox log rank test. Arrows and lines indicate the two ages (3 months and 16 months) at which mice were dissected and used for the gene expression experiments in the SAM strains. An arrow also indicates the age at which the old B6J mice were sacrificed for gene expression analysis. **(b) **Retention of foot shock is indicated as latency to entry into the dark chamber (day 2-day 1) in a passive avoidance paradigm. Difference in latency to entry is shown on the *y*-axis and age on the *x*-axis at a young time point and an old time point for S8 (blue: young *n *= 43, old *n *= 8), S10 (purple: young *n *= 31, old *n *= 7) and SR1 (green: young *n *= 41, old *n *= 27) mice. Error bars indicate standard error. S8 and S10 mice show decreased latency to entry relative to SR1 at both young (two-tailed Student's *t*-test: *p *< 0.003 and *p *< 0.002, respectively) and old (*p *< 0.05, S8) time points.

As accelerated aging of the senescence-prone mouse strains is a complex phenotype, we monitored the life span, pathology, fecundity and learning and memory behavior of all generations of mice to validate the accelerated-senescence phenotype in our facility and employed retrospective pedigree selection on S8 and S10 mice as previously described [30] (see Additional data file 2). As expected, both S8 and S10 strains had increased mortality and morbidity with age relative to SR1 and B6 mice (Figure 1a; B6 data from Pugh *et al. *[32]). S8 mice had the shortest life span, with a median life span 39% shorter than that of SR1. S10 mice showed an 18% decrease in median life span. These data are consistent with the reported life spans of SAM strains reared under conventional conditions, where the median survival time of all SAMP strains (including not only S8 and S10 but also other accelerated-senescence strains) is reported to be 40% less than that of SAMR strains [20]. Whereas the single greatest cause of death in the SAMR1 strain was cancer (consistent with the original AKR/J strain from which the SAM strains were developed), the SAMP10 and SAMP8 mice showed a decrease in cancer-related deaths and an increase in death due to infection or a wasting syndrome consistent with neurological dysfunction. Finally, an analysis of litter size also showed a significant decrease in fecundity in both S8 and S10 mice as compared with SR, consistent with accelerated aging of the reproductive organs (data not shown) [33].

Most relevant to the studies performed in our laboratory, a progressive deterioration in learning and/or memory performance has been reported in S8 and S10 mice [22,23]. To confirm and elucidate these phenotypes, behavioral analysis of our colonies was performed using a single-trial passive avoidance paradigm, in which shorter latency to entering a darkened chamber indicates a lower retention (memory) of a previous foot shock. This test was performed on both younger (average age of 16 weeks) and older (average age of 81 weeks) mice (Figure 1b). At 16 weeks of age, S8 mice demonstrate an average latency 52.1 seconds shorter than SR1 and 16 week-old S10 mice demonstrate an average latency 56.7 seconds shorter than SR1. These differences between the SAMP strains and the SAMR strain are significant using a two-tailed Student's *t*-test (*p *< 0.003 for SAMP8 versus SAMR1 and *p *< 0.002 for SAMP10 versus SAMR1). At 81 weeks of age, the retention deficits for S8 and S10 mice have worsened, with S8 mice showing a 119% decrease and S10 mice showing a 52% decrease in performance, while SR1 mice show only a 14% decrease. Consistent with previously published results, the SAMP mice exhibit a severe age-related decline in learning and memory relative to control mice. Therefore, within the cohort studied for phenotype, RNA profiling, DNA sequencing and *in situ *hybridization, the animals showed a consistent phenotype within the colony.

### Neurological gene-expression profile of aging is unique among strains

The anatomical and behavioral analyses of the SAM strains are consistent with age-related deficits in hippocampal-mediated processes that are accelerated in S8 and S10. In addition, studies of the retina also suggest a retinal degeneration phenotype specific to S8 mice that may mimic age-related declines in retinal function in humans [29]. The hippocampus and retina of old (16 month-old S8, S10, SR and 21 month-old B6J) and young (3 month-old) mice were subjected to gene-expression analysis studies using Affymetrix oligonucleotide microarrays (see Lipshutz *et al. *[31], Sandberg *et al. *[34], Caceres *et al. *[35], and Materials and methods for details). As B6J mice exhibit a longer life-span than SR1 mice, we sacrificed B6J mice at 21 months of age, approximately at the same 95% survival point as seen for 16 month-old SR1 mice (see Figure 1a). To ensure that the analysis method used minimized false positives and maximized reliability of the results, the number of independent replicate samples needed was determined by the variation inherent in the samples (see Additional data file 3). In the case of younger animals, two independent samples for each time point and tissue were required. For the 16 month-old S8, S10 and SR hippocampus samples, three samples were used. For retinal samples, four retinas from two mice were pooled to obtain sufficient RNA for each sample. Reproducibility was measured using the Pearson correlation coefficient based on the signal intensities of all genes on the array between replicate samples (perfect correlation = 1.0), and the average correlation coefficients of the replicates in each condition were as follows: young hippocampus, 0.9914; young retina, 0.9946; old retina, 0.9949; old B6J hippocampus, 0.9922, and old S8, S10 and SR hippocampus, 0.9695 (see Additional data file 3 for all replicate correlation coefficients). Representative correlation plots are shown in Additional data file 5. Whereas most of the replicates demonstrated a high reproducibility (> 0.99 correlation coefficient), there was greater variability seen in the old S8, S10 and SR hippocampus replicates (as indicated by lower correlation coefficients). As a result, in these cases additional samples were prepared and analyzed. The number of genes differentially expressed between replicates was used as an estimation of the false positive rate. In all cases very few genes were identified as differentially expressed between replicates, indicating a very low expected false-positive rate in the experimental analyses (Additional data file 3). All data and analysis tools used in this publication are available at [36].

Several types of analyses were performed. First, we characterized gene-expression profiles of aging within each strain. Pairwise comparisons between each tissue sample for young and old mice of the same strain were performed. A given mRNA transcript was considered differentially expressed in a comparison of any two samples if it met the following criteria: a Wilcoxon signed rank test (relative) (WSRR) *p*-value of *p *≤ 0.01 and increase fraction ≥ 0.7; or *p *≤ 0.0316 and increase fraction ≥ 0.8; or *p *≤ 0.01 and increase fraction ≤ 0.3; or *p *≤ 0.0316 and increase fraction ≤ 0.2. A fold change of 1.5 or greater and an average difference change in signal of 30 or more was also required. A gene was considered differentially expressed between conditions (that is, old S8 retina versus young S8 retina) only if it met the above criteria in more than 70% of the pairwise comparisons (3/4 or 4/4 comparisons), and carried a statistically significant absolute call of 'Present' (P) or 'Marginal' (M) in at least one sample (see Materials and methods for more detail).

**Figure 2:**
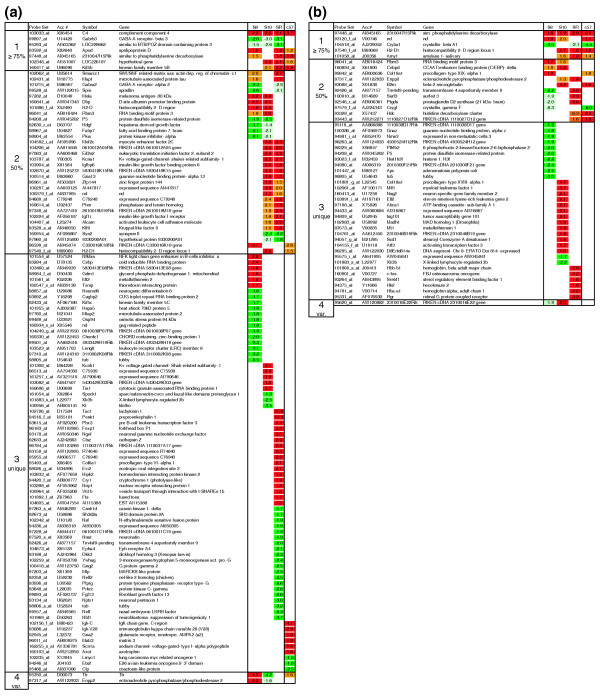
Strain-specific aging gene-expression profiles. **(a) **Heat-map view of differentially expressed genes between 3- and 16-month-old S8, S10, SR1 and B6J hippocampus. Fold change of old relative to young hippocampus is numerically indicated within each box. Bright red (increase in expression level) and bright green (decrease in expression level) indicate genes that met the most stringent criteria for an expression-level change, including an average WSRR *p *< 0.01. A gene is colored orange or light green if its expression increased or decreased with an average WSRR *p *< 0.05. Box 1, genes changed in common with three or more strains; box 2, genes changed in only two strains; box 3, genes with changes unique to a single strain; and box 4, genes with inconsistent patterns of change. **(b) **Heat-map view of differentially expressed genes between 3-month-old and 16-month-old S8, S10, SR1 and B6J retinas. The analysis was performed as described for the hippocampus.

Subsequently, those genes found to be differentially expressed by the strict criteria described above were examined in all other strains and were considered differentially expressed in another strain if the expression change during aging was significant to an average (WSRR) *p*-value = 0.05. Finally, the genes that were differentially expressed during aging in each strain were clustered into heat-map views based on their expression patterns, allowing us to examine similarities and differences in transcriptional aging between strains (Figure 2).

Unexpectedly, each strain showed a remarkably unique profile of aging. In the aging hippocampus, only a single gene out of a total 115 (complement component 4 (*C4*)) changed with age in all four strains (Figure 2a). Seven genes increased in B6J and at least one SAM strain hippocampus. Finally, two genes were downregulated with age in all three SAM strains, but did not change in B6J. The vast majority of changes during aging (75/115 or 65%) were unique to one of the four strains. Interestingly, the genetic background of the animals played an important part in the similarity of the profiles, as related SAM strains exhibited patterns of gene-expression change more similar to one another than they did to B6J, in spite of the fact that SR1 and B6J both demonstrate a 'normal' phenotype, lacking the accelerated neurological pathology seen in S8 and S10 mice.

To determine if these observations extended to other central nervous system (CNS) tissues, similar analyses of the retina were performed (Figure 2b). As seen for the hippocampal data, only a single gene (AI845165, similar to the phosphatidylserine decarboxylase gene) changed with age in all SAM strains and B6J. Also like the hippocampus, the majority (30/46 or 65%) of gene expression changes in the retina were unique to a single strain, again indicating that neuronal tissues of different strains can exhibit dramatically different transcriptional responses to aging.

### Strain differences in gene expression

The analysis of the aging retina and hippocampus demonstrated that interesting and specific transcriptional events occurred within the hippocampus and retina of each strain with age. The results suggest that differences in expression levels of important genes between the senescence-prone and -resistant strains could play an important role in mediating the age-related differences observed between these strains. One hypothesis suggests that differences in expression levels of important genes between the senescence-prone and -resistant strains could play an important role in mediating the age-related differences observed between these strains.

**Figure 3:**
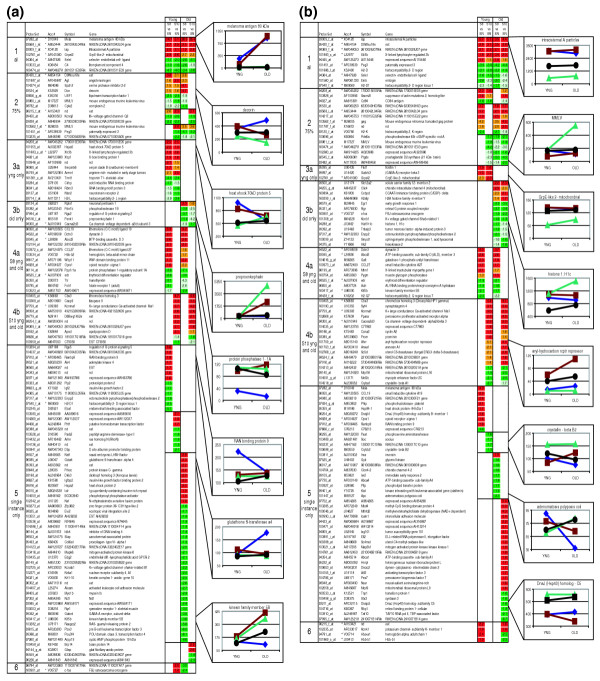
Inter-strain, age-independent gene-expression changes. **(a) **A heat-map view of genes differentially expressed in hippocampus with either S8 compared to SR1, or S10 compared to SR1 is shown. Columns indicate fold change in gene expression between young S8 and SR1, young S10 and SR1, old S8 and SR1 and old S10 and SR1, respectively. Bright red and bright green indicate genes that met the most stringent criteria for an increased or decreased expression level change, including a WSRR *p *< 0.01. A gene is colored orange or light green if it was increased or decreased with an average WSRR *p *< 0.05 in all comparison files. Boxes, numbers and examples of genes with the pattern of change are as follows: group 1, genes differentially expressed between both young and old S8 and S10 mice compared to SR1, example gene is melanoma antigen, 80 kDa; group 2, genes differentially expressed in 3/4 comparisons of young and old S8 and S10 compared to SR1, example gene is decorin; group 3, genes differentially expressed in either young and old S8 versus SR1 or young and old S10 versus SR1, example gene is heat shock 70 kD protein 5; group 4, genes differentially expressed in any single comparison group of S8 or S10 to SR1, example gene is protein phosphatase 1-1A (differences unique to a single age and strain); group 5, genes with inconsistent expression differences, example genes are RAN binding protein 9, glutathione S-transferase a4 and kinesin family member 5b. Genes differentially expressed in both retina and hippocampus are indicated by an asterisk. Gene-expression differences confirmed by qRT-PCR are indicated by the symbol ‡. Line graphs in the last column represent the signal intensity on the *y*-axis and the time point of collection on the *x*-axis. S8 is indicated by a red line, S10 by a blue line, SR1 by a green line and B6J by a black line. **(b) **Heat-map view and line graphs of exemplars from each group representing differentially expressed genes in the retina between S8 versus SR1 and S10 versus SR1 mice is shown. Analysis and representation of the data is similar as described for (a).

To identify such differences, we compared gene expression results for the senescence-accelerated S8 and S10 strains to the closely related, yet disease-free, control SR1 strain at both young and old time points (Figure 3). Gene-expression differences were identified using similar analyses to those described above, but comparing young and old S8 and S10 with SR1 (young 'prone' versus young 'resistant' or old 'prone' versus old 'resistant') (see Materials and methods and Additional data file 1 for more analysis methodology). A total of 124 genes were identified as differentially expressed in the hippocampus between strains in either young or old animals (Figure 3a). A similar analysis of the retina yielded 118 genes that differed between the senescence-prone and -resistant strains (Figure 3b).

The genes differentially expressed between the senescence-prone and -resistant strains could be early markers for aging, or may establish the foundation for accelerated senescence in the S8 and S10 strains. Several of these genes fell into interesting Gene Ontology (GO) categories, as determined by gene enrichment analysis using the GO Tree Machine [37,38]. In the hippocampus, these included genes involved in learning and behavior (phosphodiesterase 1B; Ca^2+^-calmodulin dependent, protein kinase C-gamma; and preproenkephalin 1), and genes involved in the heat-shock response (heat-shock 70 kD protein 5 (glucose-regulated protein); heat-shock protein 1B; and heat-shock protein 2). In the retina, genes fell into categories involved in the perception of light (ATP-binding cassette, subfamily A (ABC1), member 4; prominin 1 phosphodiesterase 6A, cGMP-specific, rod, alpha; and retinal G-protein-coupled receptor), chloride transport (chloride channel 4-2; gamma-aminobutyric acid (GABA-A) receptor, subunit beta 3; solute carrier family 12, member 2; and chloride intracellular channel 4 (mitochondrial)) and lipid metabolism (ATP-binding cassette, subfamily A (ABC1), member 1; ATP-binding cassette, subfamily A (ABC1), member 4; glycerol kinase; peroxisome proliferator activated receptor alpha; prostaglandin D2 synthase (brain); retinol binding protein 1, cellular; sterol-C5-desaturase (fungal ERG3, delta-5-desaturase) homolog; and sterol-C4-methyl oxidase-like).

To establish the real-world performance of both the analytical methodologies and experimental procedures used to identify gene expression changes, ten genes were chosen from the hippocampal analysis for quantitative reverse transcription PCR (qRT-PCR) verification using independent samples (from mice not used in the microarray analysis) (indicated with ‡ in Figure 3). Of the ten genes assayed, the expression changes for eight genes were confirmed with a change of 1.3-fold or greater: intracisternal-A particles (*Iap*), upstream transcription factor 1 (*Usf1*), potassium voltage-gated channel, subfamily Q, member 2 (*Kcnq2*), chemokine (C-C motif) ligand 19 (*Ccl19*), erythroid differentiation regulator (*edr*), caspase 9 (*Casp9*), chemokine (C-C motif) ligand 27 (*Ccl27*), complement and component 4 (*C4*). Of the remainder, one showed a similar trend (1.2-fold change, chromobox homolog 3 (Drosophila HP1 gamma) (*Cbx3*)) and one (ATP-binding cassette, subfamily D (ALD), member 3 (*Abcd3*)), showed no gene-expression change, and thus represents a possible false positive.

**Figure 4:**
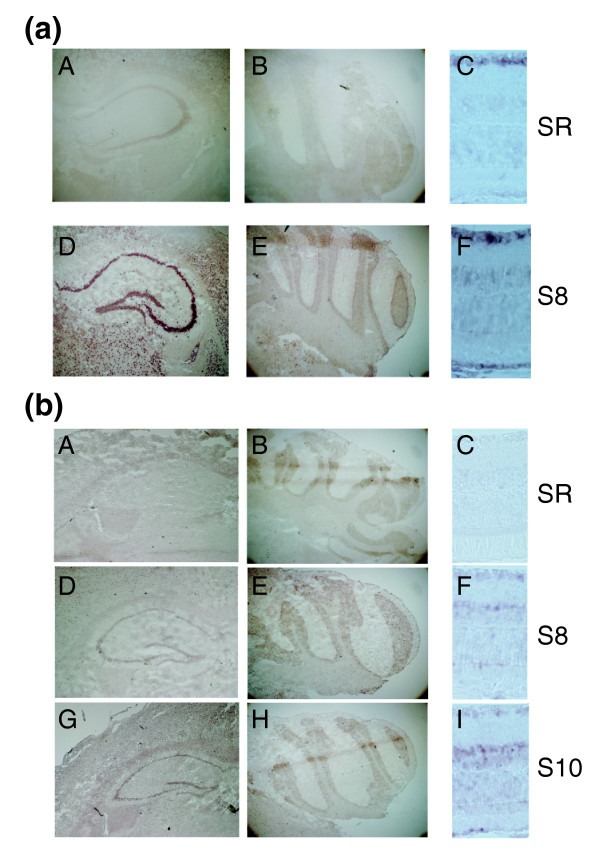
*In situ *hybridization of *Rgs5 *and *Iap*. **(a) ***In situ *hybridization of an *Rgs5 *probe to SR and S8 hippocampus (A, D), cerebellum (B, E) and retina (C, F) is shown. Increased levels of expression can be seen in all three tissues of S8 mice relative to SR1 mice. **(b) ***In situ *hybridization of an *Iap *probe to SR, S8 and S10 hippocampus (A, D, G), cerebellum (B, E, H) and retina (C, F, I) are shown. Increased expression levels of *Iap *can be seen in the hippocampus and retina of S8 and S10 mice relative to SR1 mice.

To cross-validate the gene-expression results, a method besides qRT-PCR was used to examine the expression levels of two genes identified as differentially expressed. *In situ *hybridization was performed on S8, S10, and SR1 mice for the regulator of G-protein signaling 5 (*Rgs5*) and *Iap*. Gene-expression profiling showed that *Rgs5 *was more highly expressed in the hippocampus and retina of S8 than SR1. *In situ *hybridization confirmed that *Rgs5 *was more abundant in the hippocampus and retina of S8 mice than SR1, and also revealed an increased level of *Rgs5 *in the S8 cerebellum (Figure 4a). The other transcript studied in this manner, *Iap *had a two- to five-fold higher expression level in both S8 and S10 relative to SR1 mice in the hippocampus and retina. *In situ *hybridization clearly showed an increased signal in S8 and S10 hippocampus and retina relative to SR1. In contrast to *Rgs5*, no difference was seen in the cerebellum (Figure 4b). For both transcripts, the *in situ *hybridization results were correlated with the microarray analyses, indicating a high degree of confidence in those results. Additionally, the pattern of expression observed in the *in situ *experiments suggests that the higher signal resulted from increased transcript levels in cells that normally express the gene, not ectopic expression in unusual cell types.

Using both qRT-PCR and *in situ *hybridization, 10/11 genes identified as differentially expressed using Affymetrix oligonucleotide microarrays were confirmed using independent samples and independent methods.

### A cluster of genes on chromosome 4, including *Ccl19*, is differentially expressed between S8 and SR1 mice

**Figure 5:**
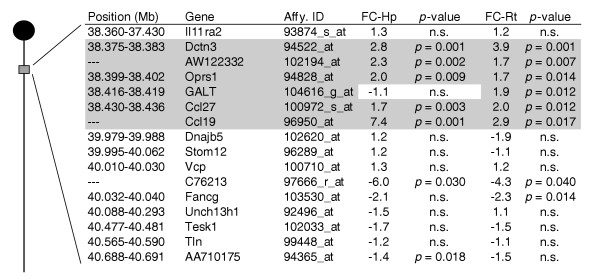
S8-specific cluster of differentially expressed genes on chromosome 4. The location and relative expression levels of a cluster of differentially expressed genes on chromosome 4 containing a cluster of differentially expressed genes in S8 mice are depicted. FC, fold change, represents the average fold change in S8 relative to SR hippocampus (Hp) and retina (Rt). Those gene-expression changes significant to a *p*-value ≤ 0.05 (WSRR) are indicated and those genes that were not significantly different are marked as nonsignificant (ns). Chromosomal map position is given in megabases, and both the Affymetrix ID and LocusLink gene symbol are specified. The genes demonstrating upregulation in S8 mice are highlighted in gray.

Because the gene-expression differences between the accelerated-senescence prone and resistant strains are consistent in multiple independent animals, we sought to identify genetic differences between the strains that might mediate these expression level differences. To identify patterns of expression related to gene position, we looked for correlations between gene location and expression difference. This analysis revealed an interesting region on chromosome 4 of S8 mice harboring multiple genes that were more highly expressed in S8 than SR1 (Figure 5). The RNA levels for six genes in retina or hippocampus were higher in S8 than SR1, representing 21% (6/29) of the S8-SR1 specific hippocampal gene expression differences and 26% (5/19) of the retinal differences. The identified genes were *Ccl19*, *Ccl27*, dynactin 3 (*Dctn3*), opioid receptor, sigma 1 (*Oprs1*), galactose-1-phosphate uridyl transferase (*Galt*), and 2810432D09Rik, all of which are within less than 100 kb of each other on chromosome 4 (based on the Celera mouse genome database). *Ccl19 *was not formally localized to this region but has been shown to be located near *Ccl27 *[39,40]. These genes are more highly expressed by a factor of 1.7 to 7.4 in S8 relative to SR1 mice. The physical clustering of differentially expressed genes may indicate involvement of a large-scale chromosomal regulatory mechanism.

**Figure 6:**
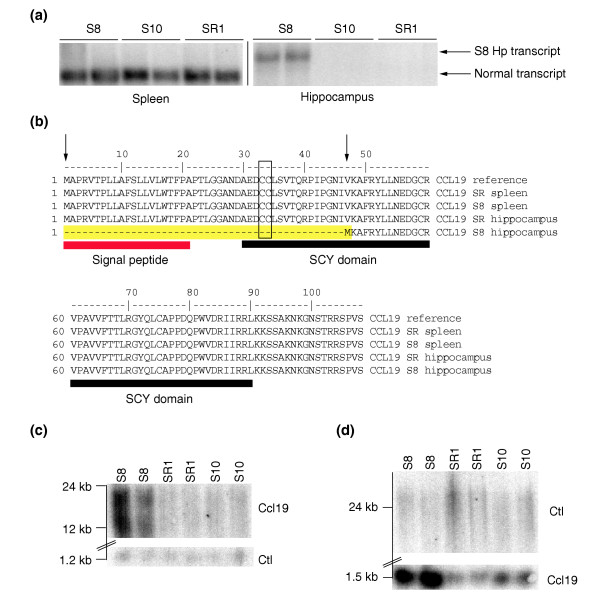
*Ccl19 *is abnormally expressed in S8 hippocampus and is duplicated. **(a) **A northern blot of total RNA from spleen and hippocampus after hybridization with a *Ccl19*-specific probe is shown. An abnormally large *Ccl19 *transcript is detected only in the hippocampus of S8 mice, while the spleen of S8, S10 and SR1 all show normal *Ccl19 *expression. **(b) **Predicted amino-acid sequence of CCL19 from the hippocampal cDNA sequence of S8 mice reveals a putative truncation at the amino terminus of the protein relative to that of S8 spleen, and SR1 spleen and hippocampus. Arrows indicate the sites of mutations in the protein found in the S8 hippocampus. The region of the protein deleted by the mutations is highlighted in yellow. A box surrounds the first two conserved cysteines, which are adjacent and conserved in all β-chemokines. Colored boxes indicate the location of the signal peptide and the SCY-domain. **(c) **A Southern blot of S8, S10 and SR1 genomic DNA digested with *Eco*RI. The upper panel shows the signal from hybridization with a *Ccl19 *probe, and the lower panel shows the signal of the same blot hybridized with a control probe demonstrating relative DNA loading. The average *Ccl19 *signal intensity of each S8 lane is 1.9-fold greater than in SR1 and S10 when normalized to the control probe (*p *< 0.005 with a two-tailed Student's *t*-test). **(d) **A Southern blot of genomic DNA digested with *Bam*HI. In this case, the lower panel shows the signal from hybridization with a *Ccl19 *probe, and the upper panel shows the signal of the same blot hybridized with a control probe demonstrating relative DNA loading. The average *Ccl19 *signal intensity seen in S8 is 2.6-fold greater than in SR1 and S10 when normalized to the control probe (*p *< 0.003 with a two-tailed Student's *t*-test).

To investigate this cluster of differentially expressed genes, we pursued one of them in more detail: *Ccl19*. Our gene-expression studies indicated an increased level of mRNA in the S8 hippocampus relative to SR1. To examine *Ccl19 *expression in the SAM strains more closely, northern analysis was performed on spleen and hippocampus RNA (Figure 6a). Whereas a consistent band was detected in the spleen of all three SAM strains (Figure 6a, lower band), a band was detected only in the hippocampus of S8 mice (consistent with the gene-expression data). Interestingly, the transcript found in the S8 hippocampus was larger than that seen in the spleen, suggesting either tissue-specific alternative splicing from a single gene locus or the presence of a novel expressed locus.

Motivated by reports that some strains of mice can harbor nearby pseudogenes of *Ccl19 *[39,40] and the different size of the transcript identified in the S8 hippocampus, sequence analysis was performed on *Ccl19 *cDNA from both hippocampus and spleen of S8, S10 and SR1 mice. While no bands were identified by northern analysis in the hippocampus of S10 or SR1, fragments were obtained from these tissues using the more sensitive method of reverse-transcriptase PCR. Sequencing of fragments amplified from cDNA revealed an altered coding sequence for the S8 hippocampus transcript relative to all other transcripts, including that found in S8 spleen. The predicted amino-acid sequence of the transcript unique to the S8 hippocampus had two mutations relative to the canonical *Ccl19 *sequence: a point mutation eliminating the canonical start ATG of *Ccl19 *and a substitution mutation resulting in a novel methionine 47 residues further downstream in the S8 hippocampus transcript (Figure 6b). Interestingly, while we found no prior description of the novel methionine, the mutation in the canonical start ATG has been previously described in unexpressed *Ccl19 *pseudogenes found in other strains of mice [39,40]. It is possible that the novel downstream ATG identified in the *Ccl19 *transcript from the S8 hippocampus may provide a compensatory in-frame start site allowing expression of a truncated protein. No differences within the coding sequence were identified that could result in the larger transcript observed in the S8 hippocampus, suggesting that the longer mRNA results from additional 5' or 3' untranslated region (UTR) sequence.

Because *Ccl19 *was one of several up-regulated genes located in proximity to one another and because sequence differences between S8 *Ccl19 *hippocampus and spleen mRNA suggested expression from at least two distinct genes, we hypothesized that a genomic duplication encompassing *Ccl19 *and surrounding genes was present within the S8 genome. Indeed, Southern analysis using a *Ccl19 *probe demonstrated a two-fold increase in signal intensity in S8 mice relative to S10 and SR1, consistent with such a duplication (Figure 6c, d). The Southern analysis and sequence information from S8 mice are consistent with a duplication of a block of genes on chromosome 4, resulting in increased expression.

### *Fgf1 *is mutated in S10 mice

As no large-scale genomic sequencing has been reported for either S10 or SR1, we used an algorithm developed in our laboratory that takes advantage of the fact that Affymetrix GeneChips use a series of oligonucleotides that span up to hundreds of bases of a given gene to detect potential sequence variations between the strains (J.A.G., M.A. Zapala, C.B. and D.J.L., unpublished data, see Materials and methods). These oligonucleotides (called probes) yield distinct patterns of intensity for each gene. Sequence differences can be detected based on differences in the hybridization pattern across the set of probes between samples. We compared the underlying patterns of signal intensity between the SAM strains to identify genes that may harbor sequence differences between strains [35] (J.A.G., M.A. Zapala, C.B.and D.J.L., unpublished data, see Materials and methods). These oligomers (probe pairs, 11-20 per gene) yield distinct patterns of intensity for each gene. The probe pairs are sensitive enough that appropriately positioned single base differences between the probe pair and the detected RNA can significantly change the signal intensity, and thus produce different patterns between slightly different sequences. We compared these underlying patterns of signal intensity between the SAM strains to identify genes harboring candidate sequence differences between strains [35] (J.A.G., M.A. Zapala, C.B. and D.J.L., unpublished data, see Materials and methods). Using a threshold *p*-value of *p *< 0.000001 (calculated from a two-tailed Student's *t*-test (unpaired, equal variance)), 20 transcripts were predicted to harbor sequence differences (possibly including nucleotide substitutions, splice differences and/or deletions/insertions) in S8, 36 genes in S10, and 17 genes in both S8 and S10 relative to SR1 (see Additional data file 4).

**Figure 7:**
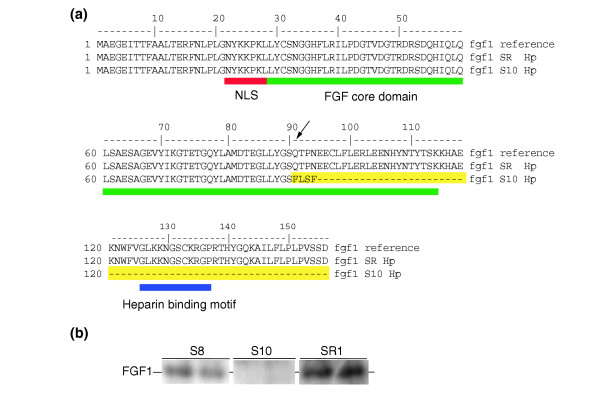
Absence of wild-type FGF1 in S10 cortex. **(a) **Predicted amino-acid sequence of FGF1 protein from SR1 and S10 hippocampus reveals a putative truncation of the S10 protein sequence at the carboxy terminus. The reference FGF1 protein sequence is shown at the top. The site of the 15-bp insertion is indicated by an arrow and the resulting amino-acid changes and the truncation predicted in S10 FGF1 are highlighted in yellow. The nuclear localization signal (NLS), the FGF core domain and the heparin-binding motif are indicated by boxes. **(b) **A western blot of S8, S10 and SR1 cortices probed with an antibody specific for the carboxy terminus of FGF1 is shown. This region is predicted to be deleted in S10 mice. Wild-type mouse FGF1 is 155 amino acids and 17.4 kD. As predicted, wild-type FGF1 is detected in the cortices of S8 and SR1, but not S10, consistent with the predicted truncation of the protein in that strain of mice.

We have found previously that genes containing at least two predicted polymorphisms (even in the 3' UTR of a gene) often contain additional sequence variations (data not shown). Therefore, we sequenced the coding regions of several genes containing predicted sequence differences between S10 and SR1 that are also known to be involved in important cellular pathways. This led to the identification of mutations in the fibroblast growth factor 1 (*Fgf1*) gene in S10 mice. Sequencing of the *Fgf1 *transcript confirmed the predicted polymorphisms in the 3' UTR in S10 (T-C at base pair 2,190 and C-T at base pair 2,931, reference sequence: AF067197). Of particular importance, further sequencing into the coding region of *Fgf1 *revealed a 15-nucleotide insertion that alters the coding sequence and is expected to result in a truncated protein lacking approximately 45% of the conserved *Fgf1 *domain (Figure 7a). To confirm that the mutation affected the protein, western blotting was performed. Western analysis on brain extracts from S8, S10 and SR1 mice using a carboxy-terminal antibody confirmed the absence of normal FGF1 protein in the brain of S10 mice (Figure 7b). As fibroblast growth factors have been linked to specific roles in the central nervous system [41,42], it is tempting to speculate that the mutation in this gene plays a role in the senescence-associated neurodegeneration seen in S10 mice.

## Discussion

Animal models for the study of complex diseases that are late-manifesting are difficult to create. This poses a major challenge when attempting to study human diseases that occur during the process of aging. This problem is exaggerated when the disease process may emerge not as a discrete phenomenon but rather as a constellation of processes that cascade and ultimately lead to the complex disease. The rare inbred mouse strains generated by careful breeding and selection known as the senescence-accelerated prone mice afforded us the opportunity to study a complex cascade that ultimately leads to progressive neurological decline during the aging process. Although these animals are difficult to maintain and little is known regarding the underlying genetics that predispose these mice to accelerated senescence, their extraordinary phenotype motivated us to study these mice using transcriptional profiling and targeted polymorphism screening to begin to dissect the pathways that lead to the disease phenotype and to compare these changes with those that occur during the 'normal' neurological aging process in two control strains.

Our studies suggest a role for the identified genes and pathways in the neuropathological phenotypes seen in S8 and S10 mice and, at a minimum, provide a set of likely candidate genes and mutations for further study. Importantly, this work demonstrates that it is possible to use large-scale gene expression profiling to identify genotypic differences between strains and link them to a phenotype. We sought to test whether gene expression profiling combined with follow-up of specific genes could be exploited to identify candidate pathways involved in aging processes. We found that we could identify genes likely to be involved in the aging process in multiple mouse strains, and also apply genomics to the study of inbred mice to proceed beyond the search for general patterns of gene expression toward the identification of specific mutations in the accelerated-aging models.

### From genomics to specific genes

The combination of gene-expression profiling and the use of inbred mouse strains revealed the presence of a block of differentially regulated genes on chromosome 4 in S8 mice. Follow-up studies confirmed a duplication event containing at least one gene in the region, *Ccl19*. Given the important role that inflammatory processes could have in CNS aging, both *Ccl19 *and the closely linked *Ccl27 *could play primary roles in the neurological phenotypes seen in the S8 strain. Our results suggest that a large-scale duplication event of a region of chromosome 4 results in increased expression of multiple genes in the brains of S8 mice.

We have also identified a potentially important mutation in the growth factor gene *Fgf1 *in S10 that results in a nonsense codon in the predicted amino-acid sequence, and western analysis confirmed that normal FGF1 protein is not detectable in the brains of these animals. Interestingly, FGF1 has been shown to protect neurons from excitotoxic stress, and low *FGF1 *mRNA levels have been implicated in Alzheimer's disease [43-46]. The absence of intact FGF1 in the S10 mice could be critical in the age-related neurodegeneration and brain atrophy observed in the S10 strain.

The opportunistic polymorphism screening approach we pursued led to multiple candidate genes. It is exciting to postulate that future availability of more complete sequence and/or high density SNP information will make possible the combination of genomic information with full genome expression profiling to delineate specific mutations and expression differences that point to a host of intriguing candidates for complex traits.

### General aspects of aging

While the majority of genes found to be differentially expressed in the hippocampus and retina were specific to one or two strains, two genes in particular stood out as potentially 'universal' markers for molecular processes involved in aging. In the hippocampus, *C4 *was upregulated in old S8, S10, SR1 and B6J mice. *C4 *was also upregulated in the aging neocortex and cerebellum of B6J, suggesting that the complement cascade plays a common role in aging in multiple inbred mouse strains and brain regions [47]. Other components of the complement cascade were also upregulated in the Lee *et al. *study [47], including C1q A-chain, C1q B-chain, and C1q C-chain. Examination of these genes in our data revealed that, while the changes were not significant enough to meet our most stringent criteria, the C1q A-chain, C1q B-chain transcripts were elevated approximately 1.3-fold in aged S8, S10 and SR1 hippocampus (S8 *p*-value = 0.05 and 0.08 for C1q A- and B-chains, respectively; S10 *p*-value = 0.03 and 0.05 for C1q A- and B-chain, respectively; and SR1 *p*-value = 0.03 and 0.1 for C1q A- and B-chains, respectively). Increases in immune-response genes in the aging brain have been found in several different mouse strains and there is a growing body of evidence supporting the importance of chronic inflammation in mammalian aging [47-49]. Intriguingly, such an immune/inflammatory response may play a particularly important role in the nervous system, as similar increases were not seen in gene expression studies of aging muscle or fibroblasts [50,51]. Other studies in B6J mice also show involvement of inflammatory pathways in the aging retina ([52] and S.Y., unpublished work). One study has also reported increased levels of immune-related transcripts with age in the liver of another strain (C3B10RF1), suggesting that there may be other tissues with age-related immune responses [53]. Importantly, studies of caloric restriction, the only known method of life-extension in mammals, show that restricting food intake results in the downregulation of genes involved in inflammation [47,49].

Another interesting gene identified was the mouse homolog of *phosphatidylserine decarboxylase *(*PSDC*), a transcript up-regulated in the aging retina of all strains and the aging hippocampus of S8, SR1 and B6J. This gene encodes an enzyme localized to the inner mitochondrial membrane that is involved in phospholipid biosynthesis. It is possible that the upregulation of *PSDC *may provide a mechanism to compensate for oxidative damage to membranes in the CNS [54]. Finally, a recent report examining gene regulation and the aging human brain identified many genes in the same pathways that we have identified here, including genes involved in stress response, inflammation and vesicular transport [55].

### Strain-specific transcriptional response to aging

In addition to the identification of genes and mutations potentially involved in aging, our results demonstrated some unexpected findings. Surprisingly, we found that different inbred strains of mice demonstrated strikingly distinct aging patterns (Figure 2). Of the many genes differentially expressed, very few were in common between the three SAM strains and B6J. More age-related gene expression changes were found in common between the SAM strains than the B6J, most likely reflecting the close genetic relationship between the AKR/J-derived SAM strains, but even within the SAM mice, there were significant differences between the strains in the patterns of expression changes. This suggests that the use of any single inbred mouse strain to study transcriptional events associated with mammalian aging may be misleading or incomplete and that it is best to use multiple strains in order to model general mammalian aging processes.

The two strains demonstrating accelerated aging, S8 and S10, shared many differences relative to SR1, including increased expression levels of stress-response genes such as those for the heat-shock proteins Hsp70-1 and Hspa5, and for X-box binding protein 1 (*Xbp1*). The senescence-prone mice also demonstrated differential expression of some genes known to be involved in human diseases: these include transthyretin (*Ttr*) and *Kcnq2*, both of which show decreased levels of expression in S8 and S10.

Another interesting gene-expression difference between the strains was the increase in Iap mRNA levels observed in S8 and S10 hippocampus and retina relative to SR1. *Iap *sequences are repetitive transposable elements present in approximately 1,000 copies per mouse genome, and transcript levels have been reported to increase with age in the liver of some strains [56-58]. This increase has been shown to be associated with demethylation of an *Iap *promoter, suggesting a failure of repression mechanisms to control retrotransposon expression in the aging mouse. In the case of S8 and S10, *Iap *expression was elevated two- to five-fold in young mice relative to SR1, and remained elevated throughout the life span of the mouse.

Interestingly, a global evaluation of transcriptional changes also revealed a difference in expression trends between S8 and the other strains. In the aging hippocampus, 61% (40/66) of differentially expressed genes were upregulated in SR1, 69% (29/42) were upregulated in S10 and 82% (14/17) were upregulated in B6J. These values are consistent with trends seen in previous studies of other regions of the B6J brain [50]. In contrast, only 46% (18/39) of genes were upregulated with age in the S8 hippocampus. These same trends were seen in the aging retina, with S10, SR1 and B6J showing upregulation of at least 80% of differentially expressed genes (22/24, 15/16 and 8/10, respectively). The aging S8 retina showed only 24% (5/21) of genes up-regulated. This general difference in age-related gene expression levels supports a global difference in transcriptional regulation in S8 neuronal tissues. Therefore, while the neurological phenotypes of S8 and S10 mice are somewhat similar, S8 mice may have a very abnormal transcriptional response to the aging process. One hypothesis suggested by the data is that certain events during aging trigger the upregulation of important genes. This response occurs in the SR1, S10 and B6J mice, but is abnormal in S8 mice. Thus, it is possible that S10 mice exhibit an acceleration of certain normal molecular responses to aging, while S8 mice exhibit a malfunction of normal transcriptional responses.

## Conclusion

Aging is a complex process involving multiple tissues and events. Many genes and pathways are implicated from our gene-expression data, with some very interesting candidates implicated in the pathology of S8 and S10 mice. Elucidation of the precise roles these candidate genes play remains difficult, but the simple identification of such candidates provides the opportunity for hypothesis formation and testing to further characterize their involvement in the aging process. Our studies suggest that careful analysis of multiple strains, multiple tissues, and the integration of gene expression data generated from multiple laboratories will be important for deciphering the molecular biology of the aging process in mammals. Ultimately, combining the knowledge gained from whole-genome sequencing of multiple strains with gene-expression analyses and careful phenotypic comparison is likely to provide great insight into the aging process.

## Materials and methods

### Animal handling

All animal procedures were performed according to protocols approved by The Salk Institute for Biological Studies Animal Care and Use Committee. C57BL/6J mice were obtained from Jackson Laboratories and were housed for one week before dissection. The senescence-accelerated mouse (SAM) strains were developed at Kyoto University from a colony of AKR/J mice that were selectively bred based on senescence, life span and pathologic phenotypes and were generously provided to our laboratory [18,20,59]. Founder mating pairs of S8/Ta (F-105), S10//Ta (F-99) and SR1TA (F-99) mice raised under specific-pathogen-free conditions were obtained from Kyoto University as approved by the SAM council. Animals were maintained in a specific-pathogen-free environment under standard 12-h light-dark cycles (lights on 6:00 AM - 6:00 PM) with food and water provided *ad libitum*. Fertility of the mice was monitored by plug-checks during matings, and fecundity was determined by recording the number of offspring. Necropsies were performed on mice that were used for dissection, euthanized, or found dead. All mice that died of natural causes were used in the pathology studies. 237 S8 mice, 169 S10 mice, and 189 SR1 mice were used for the life span analysis. Cause of death was determined by necropsy and classified as 'cancer,' 'infection,' or 'no cancer or infection.'

### Single-trial passive avoidance

Behavioral analyses were performed using a single trial in a two-compartment step-through passive avoidance apparatus [23]. During acquisition, a 0.5 mA current was applied to a floor grid for 3 sec upon animal entry into a darkened chamber. Retention was measured 24 h later as time to enter the dark chamber, up to a maximum of 300 sec. Male and female mice were tested at two time points (young time point: average age of 16 weeks; old time point: average age of 81 weeks).

### Tissue collection

Mice were sacrificed between 15:00 and 17:00 by cervical dislocation. Retinal samples were dissected first and frozen on dry ice. The brain was then removed and the hippocampus rapidly excised, followed by the removal of the spleen from the body. All samples were rapidly frozen on dry ice and stored at -80°C. Total RNA was prepared using Trizol Reagent (Invitrogen) according to the manufacturer's recommended protocol [34].

### Gene-expression profiling

Gene-expression profiling was performed on 3 month-old (young), 16 month-old (old) S8, S10 and SR1 mice, and 3 month-old and 21 month-old B6J mice. Two independent samples for each time point were used in gene-expression profiling for each strain. Because of greater replicate variability, three samples were used for hippocampus of 16-month SAM mice. A 10.0 μg sample of total RNA was used to generate labeled cRNA for each sample according to recommended protocols (Affymetrix). RNA from multiple animals was not pooled, except in the case of retina, where the retinas of two mice were combined to generate sufficient total RNA. Hybridizations were performed on MG_U74Av2 Affymetrix GeneChips for 16 h at 50°C at a final cRNA concentration of 0.66 μg/μl. Data were analyzed using the TeraGenomics relational database [36]. To identify differentially expressed genes between any two samples, a Wilcoxon signed-rank test (WSRR) *p*-value was calculated using the probe-pair difference values for a given gene between the two samples. A combined set of criteria, WSRR *p*-value of *p *≤ 0.01 and increase fraction ≥ 0.7, or *p *≤ 0.01 and increase fraction ≤ 0.3, was used to detect genes that showed an increase or decrease in expression level. To detect less robust but still potentially interesting gene expression changes, a less stringent set of criteria, *p *≤ 0.0316 and increase fraction ≥ 0.8, or *p *≤ 0.0316 and increase fraction ≤ 0.2, was used to identify genes as showing a 'marginal' increase or decrease. Additionally, a fold change of 1.5 or greater and a change in signal ≥ 30 was also required. The above analysis was performed on all possible pairwise comparisons of the samples used (that is, two independent young S8 retinal samples and two independent old S8 retinal samples resulted in four pairwise comparisons). A gene was considered differentially expressed (for example, old S8 retina vs young S8 retina) only if it met the above criteria in at least 70% of the pairwise comparisons (3/4 or 4/4 comparisons). Finally, the transcript had to meet the following criteria resulting in an absolute call of P (present, indicating the transcript reached reliably detectable levels) in at least one sample: *p *≤ 0.0316 and positive fraction ≥ 0.6, or *p *≤ 0.1 and positive fraction ≥ 0.75.

For each analysis (old vs young or strain 1 vs strain 2), all possible pairwise comparisons were generated (that is, four files resulting from a comparison of two S8 hippocampus samples to two SR, or six files resulting from a comparison of three old S10 hippocampus samples to two young S10 hippocampus samples) and used in the analyses. This analysis methodology has been described in more detail in previous publications [34,61].

The data and analysis tools are freely available at [36].

### Polymorphism prediction

Candidate genes harboring predicted polymorphisms were identified using an algorithm developed by our laboratory (J.A.G., M.A. Zapala, C.B. and D.J.L., unpublished work). Briefly, the algorithm works as follows: first, for the selected probe sets, the individual hybridization intensity values are extracted and the difference between the perfect match and the mismatch (PM-MM) intensities is calculated for each probe pair for each sample, excluding probe sets from samples that do not meet certain pattern quality measures. The PM-MM values for each of the probe sets for each sample are globally scaled (by a factor derived from the standard deviation across the multi-probe pattern obtained in each experiment) to compensate for gene-expression differences. Next, the scaled values for each sample group are averaged, and an average and a standard deviation are calculated for each probe pair in a probe set. A threshold, an empirical measure of significance, was computed for each probe pair (PP) as follows:



The algorithm was written in structured query language (SQL) using Queryman, a Teradata specific compiler [61] to run on the Teradata relational database.

### Real-time quantitative RT-PCR

RNA samples from two to three mice for each strain at each time-point were used to verify the gene-expression differences. RNA used for quantitative PCR (Q-PCR) was independent from the RNA used in the Affymetrix microarray experiments. Standard protocols were used for the generation of cDNA from RNA following elimination of genomic DNA contamination using DNA-free (Ambion). Oligonucleotide primers were designed using Applied Biosystems Primer Express software v. 1.5. ABI's SYBR Green PCR Master Mix was used for the Q-PCR reactions, which were then run on the ABI Prism 7700 sequence detection system. All Q-PCR data analysis was normalized to peptidylprolyl isomerase B (cyclophilin) levels as an internal control.

### *In situ *hybridization

Templates for probes were synthesized by PCR, subcloned into TOPO-2 (Invitrogen), linearized with *Eco*RV, and transcribed *in vitro *using digoxigenin (DIG)-labeled uridine triphosphate (Roche Biomedical Systems) according to the manufacturer's protocols. For hippocampal and cerebellar tissue preparation, mice were sacrificed by rapid cervical dislocation, the brains removed and embedded in OCT on dry ice, and sliced in 10 μm sections using a cryostat. For retinal tissue preparation, animals were perfused using 4% paraformaldehyde. The eyes were then enucleated, fixed in 4% paraformaldehyde for 24 h and embedded in paraffin and sliced. Retinal sections were rehydrated and treated with proteinase K. Subsequently, both brain and retinal sections were refixed in 4% paraformaldehyde for 10 min, washed in PBS and acetylated. Hybridization was performed overnight at 60°C using a probe concentration of 0.5-1 μg/ml, and detection was performed with anti-digoxigenin alkaline phosphatase-conjugated Fab fragments (Roche Biomedical Systems) followed by staining with NBT and BCIP (Roche Biomedical Systems). Images were collected with a Nikon microscope attached to a charge-coupled device camera and software (Media Cybernetics), using electronically acquired composite images.

### Polymorphism identification

Total RNA was extracted from hippocampus and spleen using methods described above and contaminating genomic DNA was removed using Ambion's DNA-free according to the manufacturer's recommended procedures. Primers for the amplification of regions containing putative sequence differences were designed (length = 17-26 bp; T_m _= 55-60°C). Whenever possible, primers were designed to include the complete Affymetrix target sequence. All sequencing was performed by the Salk Institute Sequencing Core.

### Northern analysis

Total RNA (10.0 μg for each sample) was incubated at 50°C for 1 h in the presence of glyoxal, and electrophoresed in 1× DEPC-treated BPTE buffer in a 1% agarose gel. The gel was blotted onto Hybond-N+ membrane overnight. A fragment of the *Ccl19 *mRNA was amplified using RT-PCR from hippocampus total RNA. Random primed labeling was used to radioactively label the fragment with ^32^P and hybridization to the membrane was performed overnight in Church buffer at 65°C. Four washes of 15-20 min were performed to a maximal stringency of 0.1× SSC, 0.1% SDS at 65°C. Visualization was performed on a Molecular Dynamics Phosphorimaging system.

### Southern analysis

Genomic DNA (10.0 μg for each sample) was digested at37°C using *Bam*HI or *Eco*RI. The digested samples were electrophoresed in 1× TAE buffer on a 0.8% agarose gel overnight at 30 V. The gel was then washed in 0.2 M HCl for 30 min., rinsed quickly in distilled water twice, placed in denaturing buffer (1.5 M NaCl, 0.5 M NaOH) for 30 min, neutralization buffer (1.5 M NaCl, 0.5 M Tris pH 7.5) for 30 min, and 20× SSC for 2 min. The gel was then blotted onto a Nytran supercharge filter (Schleicher & Schuell) in 20× SSC overnight and UV cross-linked. To create the *Ccl19 *probe, a fragment of the *Ccl19 *mRNA was amplified using RT-PCR from SR cortex total RNA (primers: GCGGGCTCACTGG-GGCACAC, TGGGAAGGTCCAGAGAACCAG). Random primed labeling was used to radioactively label the fragment with ^32^P and hybridization to the membrane was performed overnight in Church buffer at 65°C. Four washes of 15-20 min were performed to a maximal stringency of 0.1× SSC, 0.1% SDS at 55°C. Visualization was performed on a Molecular Dynamics Phosphorimaging screen. A *Sac*I-*Eco*RI 795-bp fragment of *Grik1 *was subsequently used as a control probe following the procedures described for *Ccl19*.

### Immunoprecipitation and western blot

Cortices for two mice of each strain were homogenized in NP-40 lysis Buffer (20 mM Tris-HCl pH 8.0, 137 mM NaCl, 10% glycerol, 1% Nonidet P-40, 1 mM EDTA, protease inhibitors (aprotinin + leupeptin), 1 mM phenyl methyl sulfonyl fluoride (PMSF)) at 4°C. The samples were then centrifuged and the supernatant was removed and stored. The protein supernatant (1 mg) was pre-cleared with Protein-G Sepharose slurry for 30 min, then removed and incubated with the Protein-G Sepharose for 2 h with the anti-FGF1 antibody raised against the carboxy terminus of human FGF1 (Santa Cruz Biotechnology, 1/100 concentration). The Protein-G Sepharose beads were then removed, washed, and the protein was liberated upon incubation with SDS buffer at 70°C for 10 min. The supernatant was then run out on a Novex 12% Bis-Tris gel in MOPS buffer, blotted, and visualized with a secondary horseradish peroxidase-conjugate antibody and detected using the ECL Plus kit (Amersham Biosciences).

## Additional data files

The following additional data are available with the online version of this paper.  Additional data file 1 contains four sections explaining the animal handling and gene expression methodologies. Additional data file 5 contains four correlation plots of replicate samples. Additional data file 2 contains a summary of all mice used for breeding. Additional data file 3 contains a summary of the correlations between all replicate samples and the empirically determined estimated false-positive rates. Additional data file 4 contains a list of the genes predicted to harbor polymorphisms between the S8, S10 and SR1 strains.

## Supplementary Material

Additional data file 1 Animal handling and gene expression methodologies. Click here for file

Additional data file 2 A table containing a summary of all mice used for breeding Click here for file

Additional data file 3 A table containing a summary of the correlations between all replicate samples and the empirically determined estimated false positive rates. Click here for file

Additional data file 4 A table listing the genes predicted to harbor polymorphisms between the S8, S10 and SR1 strains. Click here for file

Additional data file 5 Correlation plots for independent replicate measurements. Examples shown are representative of the gene expression data quality obtained throughout the study. The signal for every gene on the microarray is graphed on a scatter plot for two replicate samples (*that is*, samples of the same strain, same tissue and same age but different animals). The Pearson correlation coefficient (R) is given for each comparison, ranging from 0.9752 to 0.9958. (A) Correlation plot for S8: 609-4HpOld and S8: 609-3HpOld showing the high degree of correlation between the two independent replicates. (B) Correlation plot for one of the same samples as in (A) (S8: 609-3HpOld) and a third replicate sample (S8: 674-2HpOld) showing a larger variability between replicates. (C) Correlation plot for S10: 704-3+704-4 Retina Old and S10: 15-4+705-2 Retina Old. (D) Correlation plot for SR1: 1349-1+1349-2 Retina Young and SR1: 1348-1+1348-2. Click here for file
