# Supplementary material for: Mechanisms of aging in senescence-accelerated mice
Source: Genome Biol. 2005 Jun 1;6(6):R48. doi: 10.1186/gb-2005-6-6-r48 (PMC1175968; doi:10.1186/gb-2005-6-6-r48)
Supplement: Additional data file 1 — Animal handling and gene expression methodologies. [file gb-2005-6-6-r48-S1.doc]

**Supplemental Information**

**Mouse Colony Maintenance**

The S8 and S10 strains require selective to maintain phenotypic stability within a colony. As the phenotype of interest involved late-manifesting senescence-related traits, the data necessary for selection was typically obtained after the mice had lost reproductive ability. Therefore, we employed a retrospective pedigree selection methodology previously described for the creation and maintenance of the SAM strains to ensure retention of accelerated neurological senescence in our colonies[1]. This was accomplished by following the phenotype of many breeding pairs through senescence, followed by the continuous selection of offspring of the breeding pairs that exhibited the desired traits. Additionally, we avoided sibling crosses to prevent the fixing of independent traits, and typically bred together offspring of different breeding pairs that exhibited accelerated senescence. While this required unusually large colonies and phenotyping efforts, it appears to be the most effective way of maintaining the complex (and therefore interesting) neurological phenotypes exhibited by the S8 and S10 strains[1].

**Gene Expression Profiling**

Data Access

All gene expression data used in this manuscript is publicly available along with the gene expression software analysis tools in the MIAME-compliant Teragenomics database ([http://www.teragenomics.com/public/researchNews_publications.asp](http://www.teragenomics.com/reviewers) ).

Quality Control

Sample quality can be determined by the comparison of independent replicate samples, and the correlation coefficient between signal intensities of two replicates can be used as a quantitative measure of the differences between those samples. A correlation coefficient of 1.0 indicates perfect correlation, with correlation coefficients R > 0.98 indicating two samples with very high reproducibility. A more direct measurement of the false positive rate inherent within sample data is to directly compare two replicate samples and measure the number of genes that meet the criteria for being differentially expressed between them. As the samples are replicates of one another, there should theoretically be no genes differentially expressed between them. To determine our estimated false positive rates, we examined the number of genes differentially expressed between all replicate samples in our studies (**see Supplemental Table 1**) and analyzed the replicate samples in a manner similar to that of the true comparisons. The low number of false positives (0-1 genes) indicated that the criteria used were appropriate for experimental analyses.

Gene Expression Analytic Methods

Two important statistically derived metrics used in our gene expression analysis are the Absolute Call and the Difference Call. These are described in detail below:

*Absolute Call*

The absolute call is designed to provide a qualitative determination of whether there is clear evidence that a specific mRNA is present in the hybridization sample. This determination is based on the observed hybridization pattern across all the probes in a probe set, and is meant to indicate whether the mRNA is clearly detectable (Present) or not (Absent). The call algorithm uses the collection of (PM-MM) values across a probe set, and is based on the p-value (paired, two-tailed) calculated using the non-parametric Wilcoxon signed rank test (relative) as well as the “positive fraction” (PF, the fraction of PM-MM values that are greater than zero). The call thresholds were set to maximize sensitivity to low abundance mRNAs while keeping the false positive rate to a minimum, and were based on an analysis of a large amount of experimental data.

Present (P):

p ≤ 0.0316 AND positive fraction ≥ 0.6 OR p ≤ 0.1 AND positive fraction ≥ 0.75

Marginal (M):

0.0316 < p ≤ 0.1 AND 0.6 ≤ positive fraction < 0.75 OR not called P AND positive fraction > 0.75

Absent (A):

All other probe sets that do not fall into the above categories are given a call of absent (A).

*Difference Call*

The difference call is designed to provide a qualitative determination of whether there is an expression level difference between two measurements. The metrics used are analogous to those used for the absolute call. The difference call is based on differences in the observed hybridization patterns (scaled intensities) between two measurements for all the probes in a probe set. The algorithm uses the collection of matched differences between the appropriately scaled PM-MM values (experiment 1 compared to experiment 2), and is again based on the p-value (paired, two-tailed) calculated using the non-parametric Wilcoxon signed rank test (relative) as well as the “increase fraction” (the fraction of (PM-MM)_1_ – (PM-MM)_2_ values that are greater or less than zero).

In particular, the algorithm uses the collection of relative differences for each probe pair in the probe set:

{((PM – MM)_1_ – (PM – MM) _2_) / (|(PM – MM) _1_| + |(PM – MM) _2_|)}

Absolute values are used in the denominator because PM-MM values can be negative, and a minimum denominator setting is used to avoid dividing by zero or a number that is too small relative to the noise.

In addition, to make the difference call even more conservative (and to minimize the effects of small inter-chip scaling imperfections), the difference p-value for each probe set is calculated multiple times under the assumption that the sca1ing factor could be off by as much as a factor of 1.15 in either direction. Following this calculation, the largest, or most conservative p-value is used to make the difference call (and is the p-value reported for other uses). The difference call thresholds were set to maximize sensitivity to small expression differences while keeping the false positive rate to a minimum, and were based on an analysis of a large amount of experimental data.

Increase (I):

p ≤ 0.01 AND increase fraction ≥ 0.7

Marginal Increase (MI):

p ≤ 0.0316 AND increase fraction ≥ 0.8

Decrease (D):

p ≤ 0.01 AND increase fraction ≤ 0.3

Marginal Decrease (MD):

p ≤ 0.0316 AND increase fraction ≤ 0.2

1. Hosokawa M, Abe T, Higuchi K, Shimakawa K, Omori Y, Matsushita T, Kogishi K, Deguchi E, Kishimoto Y, Yasuoka K, Takeda T**: Management and design of the maintenance of SAM mouse strains: an animal model for accelerated senescence and age-associated disorde**rs*. Exp Geronto*l 1997**,** 32:111-116.

| Supplemental Table I: SAM Colony Management | | |  |  |
| --- | --- | --- | --- | --- |
|  |  |  |  |  |
| **A. Samp8/Ta** |  |  |  |  |
| **Generation** | **Total Number of Animals** | **Number of Breeding Pairs** | **Number (%) Tested for Behavior** | **Number (%) of litters evaluated for fecundity** |
| F-105 | 7 | 7 | 3 (43) | 14 (88) |
| F-106 | 67 | 18 | 28 (42) | 19 (76) |
| F-107 | 84 | 26 | 35 (42) | 12 (39) |
| F-108 | 74 | 16 | 45 (61) | 10 (77) |
| F-109 | 51 | 6 | 28 (55) | 5 (83) |
| F-110 | 20 | 0 | 6 (30) | n/a |
| **Total** | **303** | **73** | **145 (48)** | **60 (66)** |
|  |  |  |  |  |
| **B. Samp10//Ta** |  |  |  |  |
| **Generation** | **Total Number of Animals** | **Number of Breeding Pairs** | **Number (%) Tested for Behavior** | **Number (%) of litters evaluated for fecundity** |
| F-99 | 7 | 4 | 3 (43) | 10 (83) |
| F-100 | 47 | 17 | 20 (43) | 13 (81) |
| F-101 | 59 | 16 | 29 (49) | 10 (67) |
| F-102 | 28 | 8 | 18 (64) | 4 (57) |
| F-103 | 25 | 9 | 24 (96) | 7 (70) |
| F-104 | 45 | 0 | 21 (47) | n/a |
| **Total** | **211** | **54** | **115 (55)** | **44 (73)** |
|  |  |  |  |  |
| **C. SAMR1TA** |  |  |  |  |
| **Generation** | **Total Number of Animals** | **Number of Breeding Pairs** | **Number (%) Tested for Behavior** | **Number (%) of litters evaluated for fecundity** |
| F-99 | 7 | 7 | 2 (29) | 12 (75) |
| F-100 | 71 | 12 | 39 (55) | 11 (79) |
| F-101 | 67 | 9 | 45 (67) | 5 (33) |
| F-102 | 12 | 10 | 11 (92) | 5 (83) |
| F-103 | 8 | 11 | 8 (100) | n/a |
| F-104 | 28 | 12 | 28 (100) | 6 (100) |
| F-105 | 15 | 0 | 15 (100) | n/a |
| **Total** | **208** | **61** | **148 (71)** | **39 (68)** |
|  |  |  |  |  |
| Notes on procedure: | |  |  |  |
| >Total number of mice excludes those from litters that were eaten, of unknown size, sacked at weaning, or used for mouse embryonic fibroblast production. | | | | |
| >The youngest generation of the breeding pair for mice of different generations was used for determining to which generation a breeding pair belonged. | | | | |
| >Fecundity evaluation excludes litters of unknown size which were sacked upon weaning, eaten, or used for mouse embryonic fibroblast production. | | | | |
| >The generation assigned to the litters evaluated for fecundity is determined by the mother of litter. | | | | |

| Supplemental Table II: Gene Expression False Positive Determination | | | | | |
| --- | --- | --- | --- | --- | --- |
|  | | | | | |
|  |  | R-value | # false positives |  |  |
| Hippocampus | S8 Hp young 1 vs. 2 | 0.9938 | 1 gene | The following criteria were used to determine the number of false positives: | |
|  | S10 Hp young 1 vs. 2 | 0.9923 |  |  |  |
|  | SR1 Hp young 1 vs. 2 | 0.9914 |  |  |  |
|  | S8 Hp old 1 vs. 2 | 0.9905 |  |  |  |
|  | S8 Hp old 1 vs. 3 | 0.9723 |  | Fold Change ≥ 1.5; Difference Call: I/D/MI/MD; and a signal difference ≥ 30, in ≥ 70% of comparisons; and an Absolute Call of P or M in at least one file | |
|  | S8 Hp old 2 vs. 3 | 0.9606 |  |  |  |
|  | S10 Hp old 1 vs. 2 | 0.9510 |  |  |  |
|  | S10 Hp old 1 vs. 3 | 0.9597 |  |  |  |
|  | S10 Hp old 2 vs. 3 | 0.9765 |  |  |  |
|  | SR1 Hp old 1 vs. 2 | 0.9616 |  |  |  |
|  | SR1 Hp old 1 vs. 3 | 0.9628 |  |  |  |
|  | SR1 Hp old 2 vs. 3 | 0.9903 |  |  |  |
|  | B6J Hp yng 1 vs. 2 | 0.9879 |  |  |  |
|  | B6J Hp old 1 vs. 2 | 0.9922 |  |  |  |
| Retina | S8 Ret young 1 vs. 2 | 0.9927 | 0 genes |  |  |
|  | S10 Ret young 1 vs. 2 | 0.9958 |  |  |  |
|  | SR1 Ret young 1 vs. 2 | 0.9958 |  |  |  |
|  | S8 Ret old 1 vs. 2 | 0.9975 |  |  |  |
|  | S10 Ret old 1 vs. 2 | 0.9952 |  |  |  |
|  | SR1 Ret old 1 vs. 2 | 0.9968 |  |  |  |
|  | B6J Ret yng 1 vs. 2 | 0.9942 |  |  |  |
|  | B6J Ret old 1 vs. 2 | 0.9901 |  |  |  |
|  |  |  |  |  |  |
| **Absolute Call**: This metric is used to determine whether a specific mRNA is present in the sample.). The call algorithm uses the collection of (PM-MM) values across a probe set, and is based on the p-value (paired, two-tailed) calculated using the non-parametric Wilcoxon signed rank test (relative) as well as the “positive fraction” (PF, the fraction of PM-MM values that are greater than zero) | | | | | |
|  |  |  |  |  |  |
|  |  |  |  |  |  |
|  |  |  |  |  |  |
|  |  |  |  |  |  |
| Absolute Call | | Criteria | | | |
| P (present) | | p <= 0.0316 AND positive fraction >= 0.6 | | | |
|  |  | OR | | | |
|  |  | p <= 0.1 AND positive fraction >= 0.75 | | | |
| M (marginal) | | 0.0316 < p <= 0.1 AND 0.6 <= positive fraction < 0.75 | | | |
|  |  | OR | | | |
|  |  | not called P AND positive fraction >= 0.75 | | | |
| RP (reverse present) | | p <= 0.0316 AND positive fraction <= 0.4 | | | |
|  |  | OR | | | |
|  |  | p <= 0.1 AND positive fraction <= 0.25 | | | |
| A (absent) | | All other probe sets that do not fall into the above categories are given a call of absent (A). | | | |
|  |  |  |  |  |  |
|  |  |  |  |  |  |
|  |  |  |  |  |  |
| **Difference Call**: This metric is used to determine whether a specific mRNA is present in the sample. The difference call is based on differences in the observed hybridization patterns (scaled intensities) between two measurements for all the probes in a probe set. The algorithm uses the collection of matched differences between the appropriately scaled PM-MM values (experiment 1 compared to experiment 2), and is again based on the p-value (paired, two-tailed) calculated using the non-parametric Wilcoxon signed rank test (relative) as well as the “increase fraction” (the fraction of (PM-MM)1 – (PM-MM)2 values that are greater than zero). | | | | | |
|  |  |  |  |  |  |
|  |  |  |  |  |  |
|  |  |  |  |  |  |
|  |  |  |  |  |  |
|  |  |  |  |  |  |
|  |  |  |  |  |  |
|  |  |  |  |  |  |
| Difference Call | | Criteria | | | |
| I (increase) | | p <= 0.01 AND increase fraction >= 0.7 | | | |
| MI (marginal increase) | | p <= 0.0316 AND increase fraction >= 0.8 | | | |
| D (decrease) | | p <= 0.01 AND increase fraction <= 0.3 | | | |
| MD (marginal decrease) | | p <= 0.0316 AND increase fraction <= 0.2 | | | |
