# Supplementary figures and images for: Mechanisms of aging in senescence-accelerated mice
Source: Genome Biol. 2005 Jun 1;6(6):R48. doi: 10.1186/gb-2005-6-6-r48 (PMC1175968; doi:10.1186/gb-2005-6-6-r48)

Supplemental Figure 1

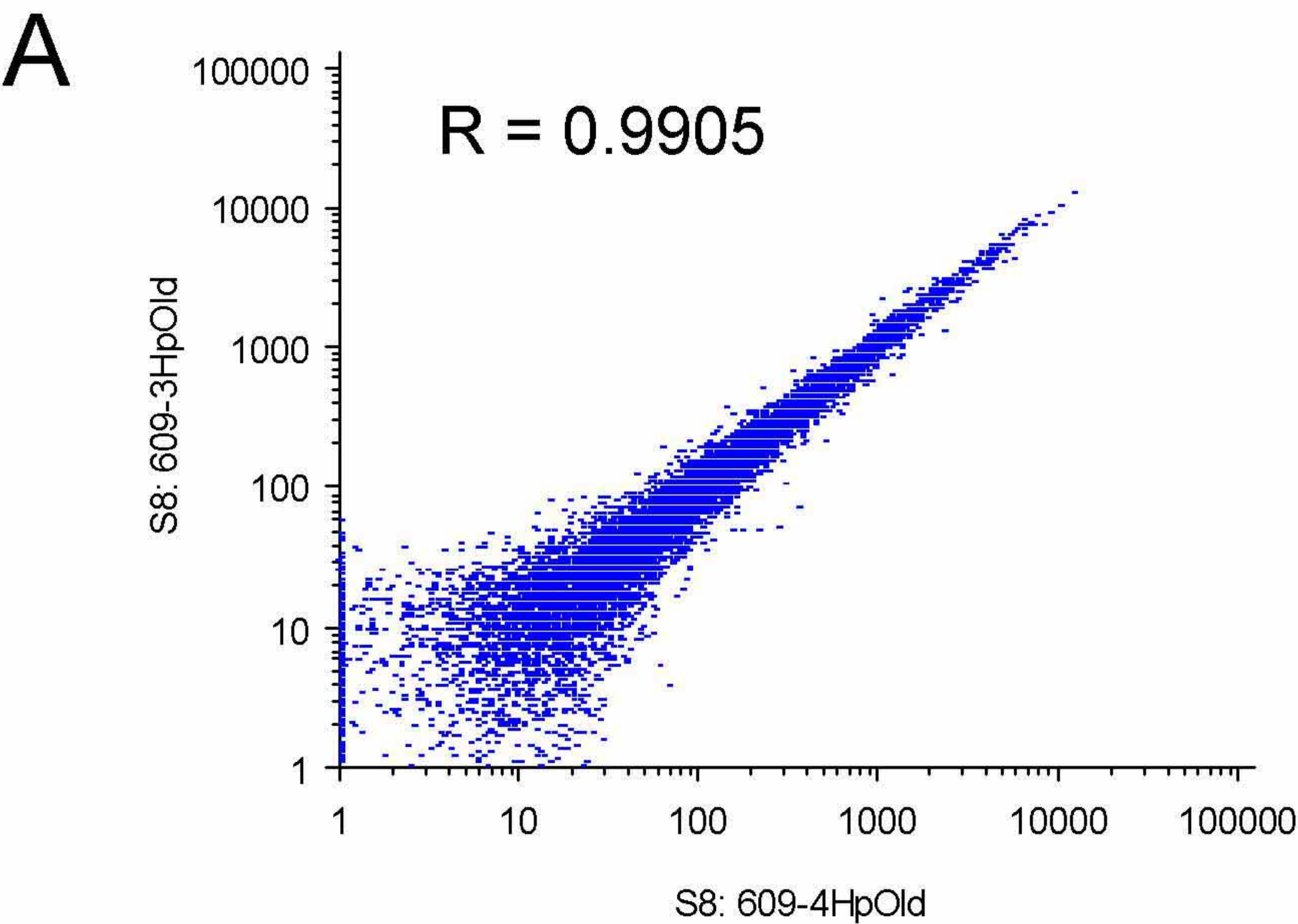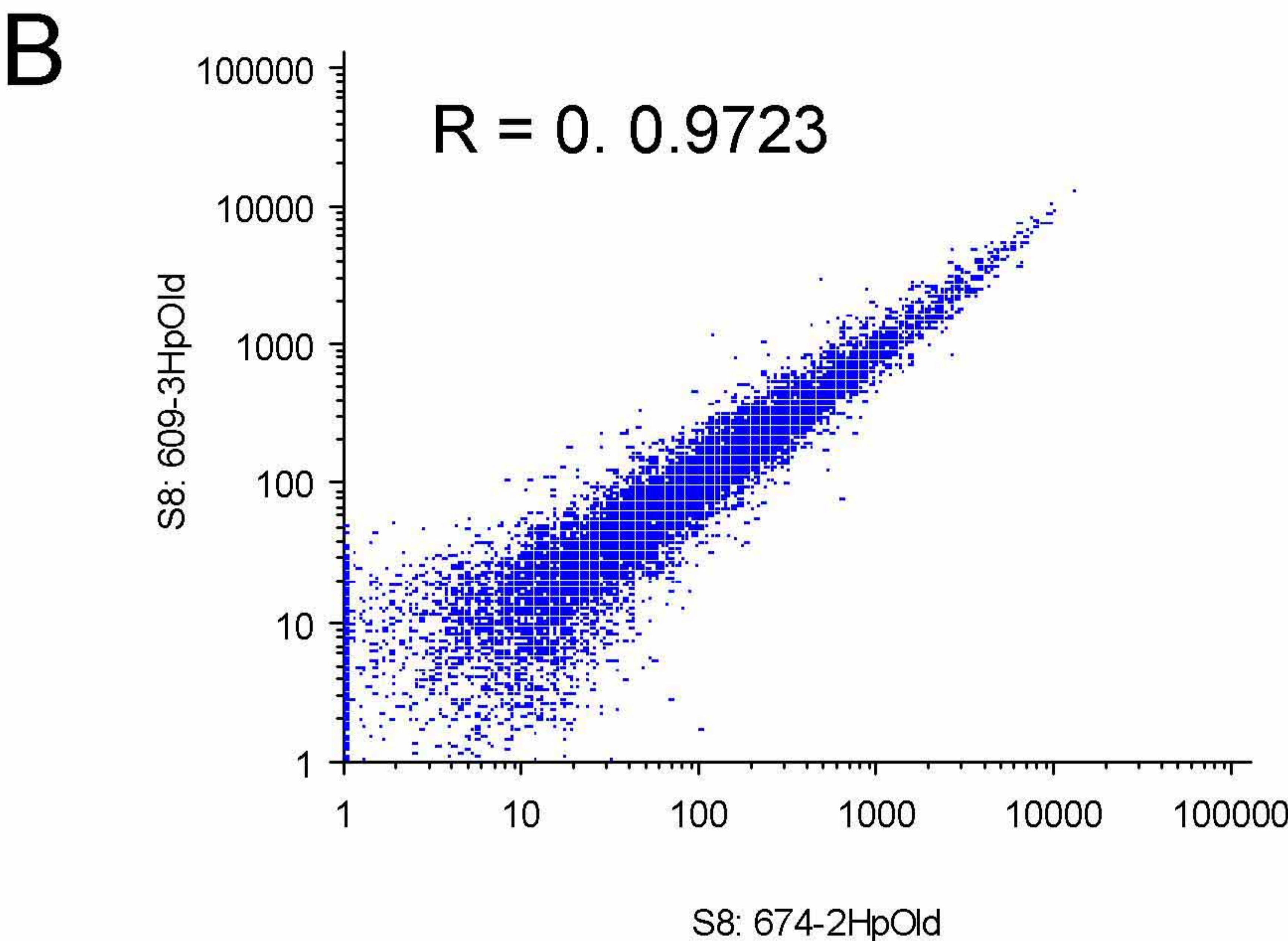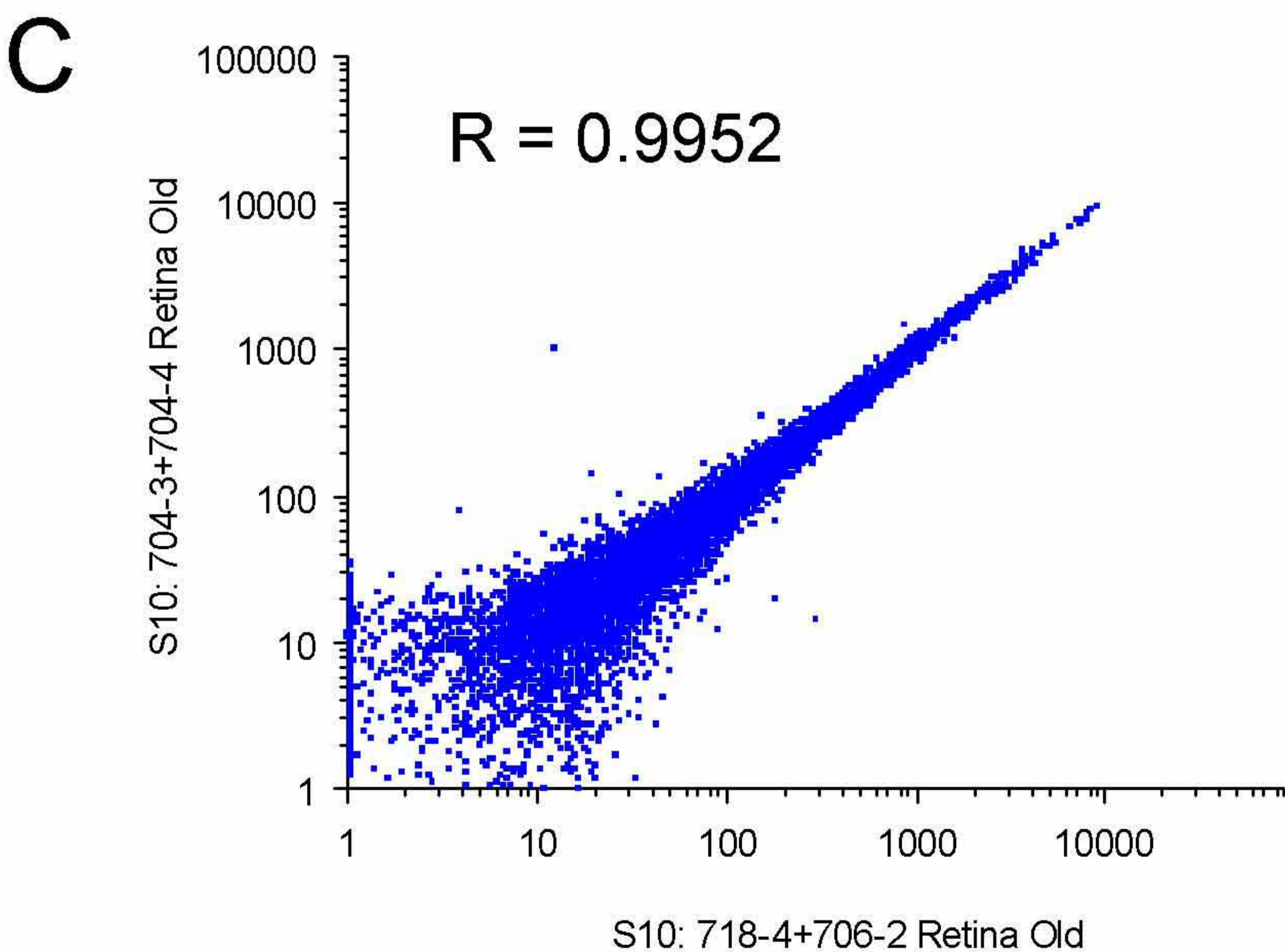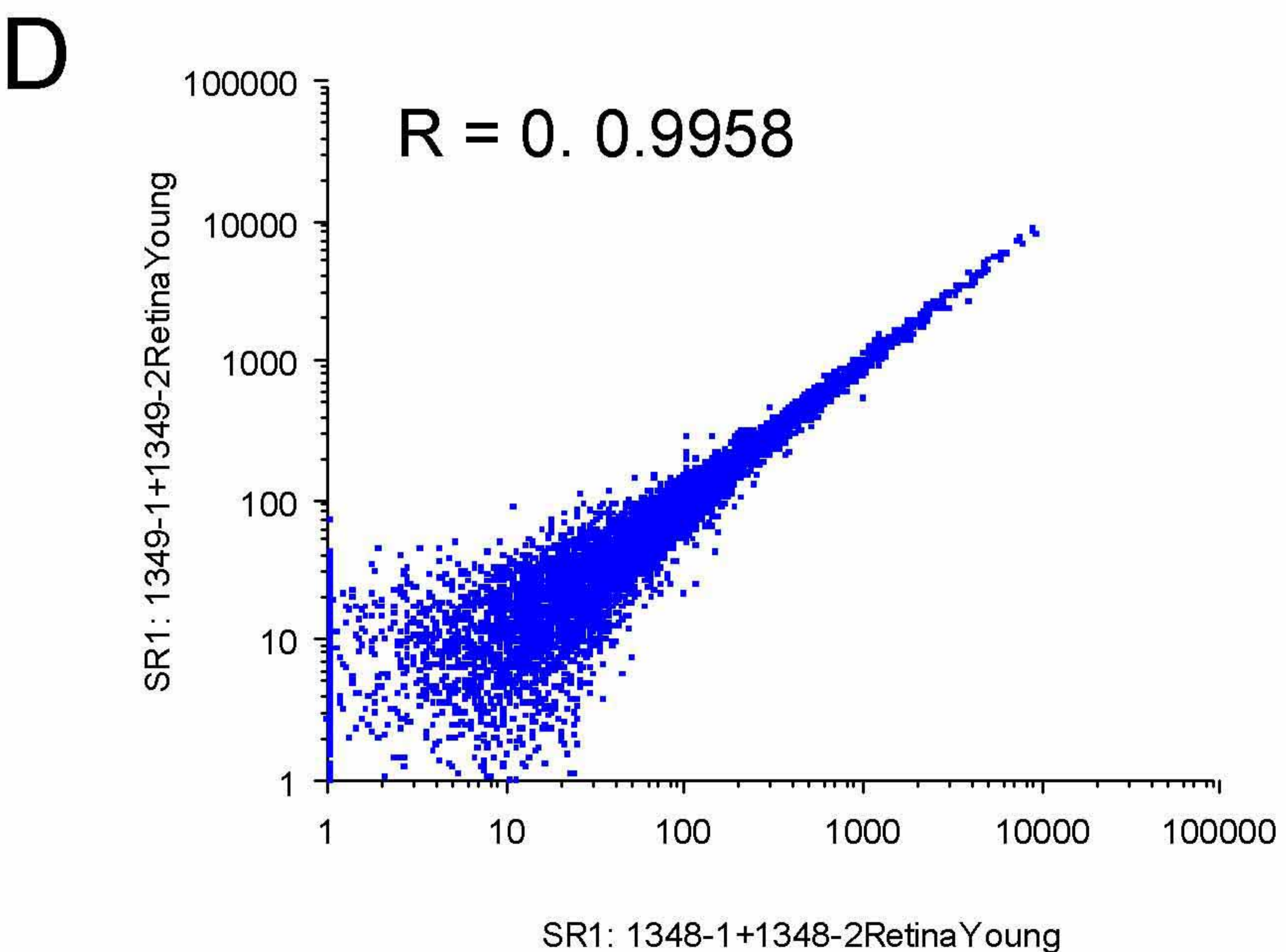

Supplement: Additional data file 5 — Correlation plots for independent replicate measurements. Examples shown are representative of the gene expression data quality obtained throughout the study. The signal for every gene on the microarray is graphed on a scatter plot for two replicate samples (that is, samples of the same strain, same tissue and same age but different animals). The Pearson correlation coefficient (R) is given for each comparison, ranging from 0.9752 to 0.9958. (A) Correlation plot for S8: 609-4HpOld and S8: 609-3HpOld showing the high degree of correlation between the two independent replicates. (B) Correlation plot for one of the same samples as in (A) (S8: 609-3HpOld) and a third replicate sample (S8: 674-2HpOld) showing a larger variability between replicates. (C) Correlation plot for S10: 704-3+704-4 Retina Old and S10: 15-4+705-2 Retina Old. (D) Correlation plot for SR1: 1349-1+1349-2 Retina Young and SR1: 1348-1+1348-2. [file gb-2005-6-6-r48-S5.pdf]
